# A Case of Retroperitoneal Paraganglioma Presenting As Dizziness and Chest Pain With a Coexisting Contralateral Primary Renal Tumor

**DOI:** 10.7759/cureus.37793

**Published:** 2023-04-18

**Authors:** Po-Hua Chen, Nimisha Madas, Farah Zahra

**Affiliations:** 1 Internal Medicine, Chicago Medical School Internal Medicine Residency Program at Northwestern Medicine McHenry Hospital, McHenry, USA

**Keywords:** community healthcare, atypical chest pain, catecholamine hypersecretion, renal oncocytoma, retroperitoneal paraganglioma

## Abstract

Paraganglioma is a rare type of neuroendocrine tumor with variable clinical presentations, making diagnosis relatively challenging. In this report, we present a case of retroperitoneal paraganglioma in a patient who experienced intermittent episodes of dizziness and chest pain. Imaging studies conducted during the patient's hospitalization revealed the presence of a lesion in the upper region of the right kidney, as well as a mass in the left retroperitoneal area that was suspected to be a paraganglioma. Biochemical studies were collected, including 24-hour urine metanephrines, urine catecholamines, urine cortisol, plasma metanephrines, renin, and aldosterone. However, it took an extended period of time for these results to come back. Given high clinical suspicion, alpha-blockade was initiated without a definite diagnosis of paraganglioma. Ultimately, the patient underwent tumor resection and the final pathology confirmed paraganglioma. The pathology of the contralateral renal mass showed oncocytoma. This case serves as an illustration of the difficulties that can arise when diagnosing and treating undiagnosed paragangliomas within a community healthcare setting.

## Introduction

Paragangliomas are uncommon neuroendocrine tumors that originate from extra-adrenal paraganglia [1]. In the United States, the estimated annual incidence of pheochromocytoma and paraganglioma ranges from 500 to 1600 cases per year [2]. The clinical presentation of paragangliomas can vary greatly depending on the location of the tumor and its secretory activity, with headaches, palpitations, and sweating being the most commonly reported symptoms [1]. A diagnosis of paraganglioma usually requires the measurement of urinary metanephrines, urine catecholamines, and/or plasma metanephrines [3]. In this report, we present a case of retroperitoneal paraganglioma that initially presented with dizziness and chest pain, along with a concurrent contralateral renal mass. We also discuss the clinical factors that influenced subsequent medical management.

## Case presentation

**Figure 1:**
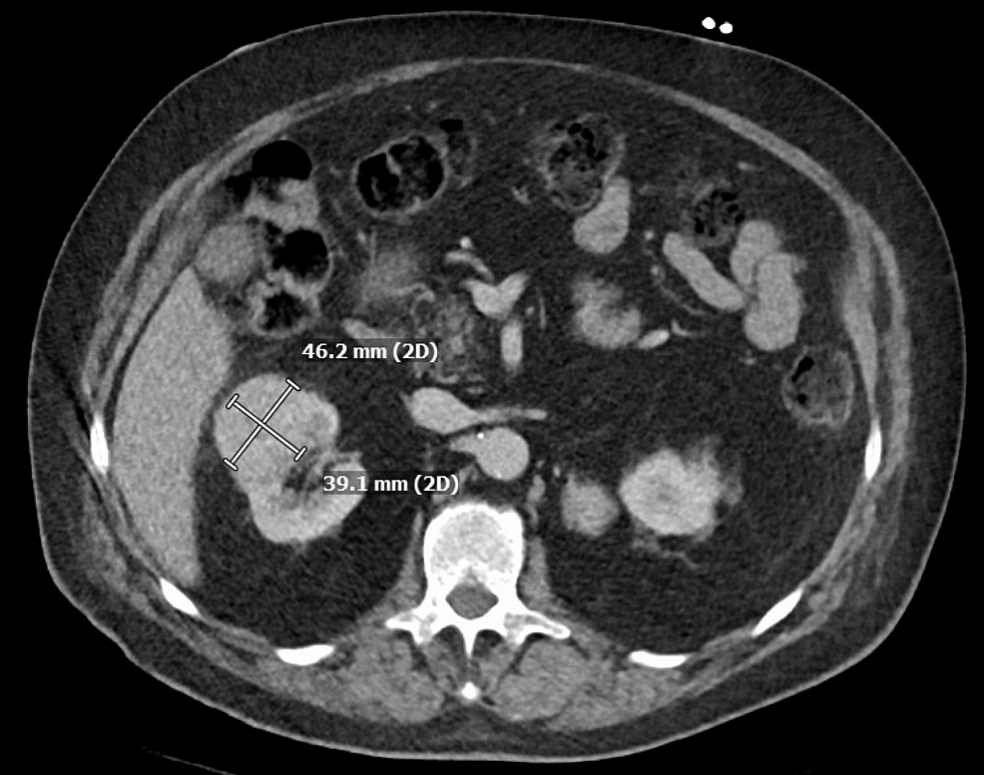
CT abdomen pelvis demonstrated a 4.6 x 3.9 x 4.3 cm right upper renal lesion. CT: computed tomography

**Figure 2:**
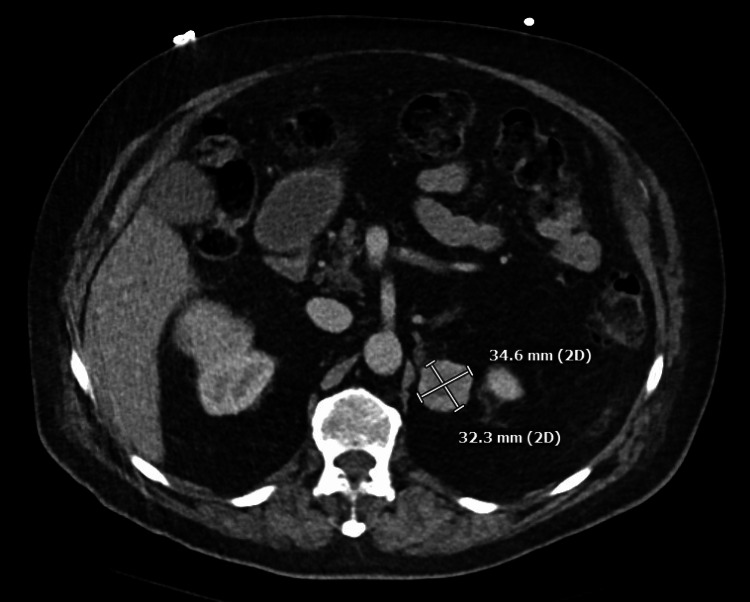
CT abdomen pelvis demonstrated a 3.5 x 3.2 x 3.4 cm soft tissue lesion adjacent to the superior pole of the left kidney and posterior to the left adrenal gland. CT: computed tomography

**Figure 3:**
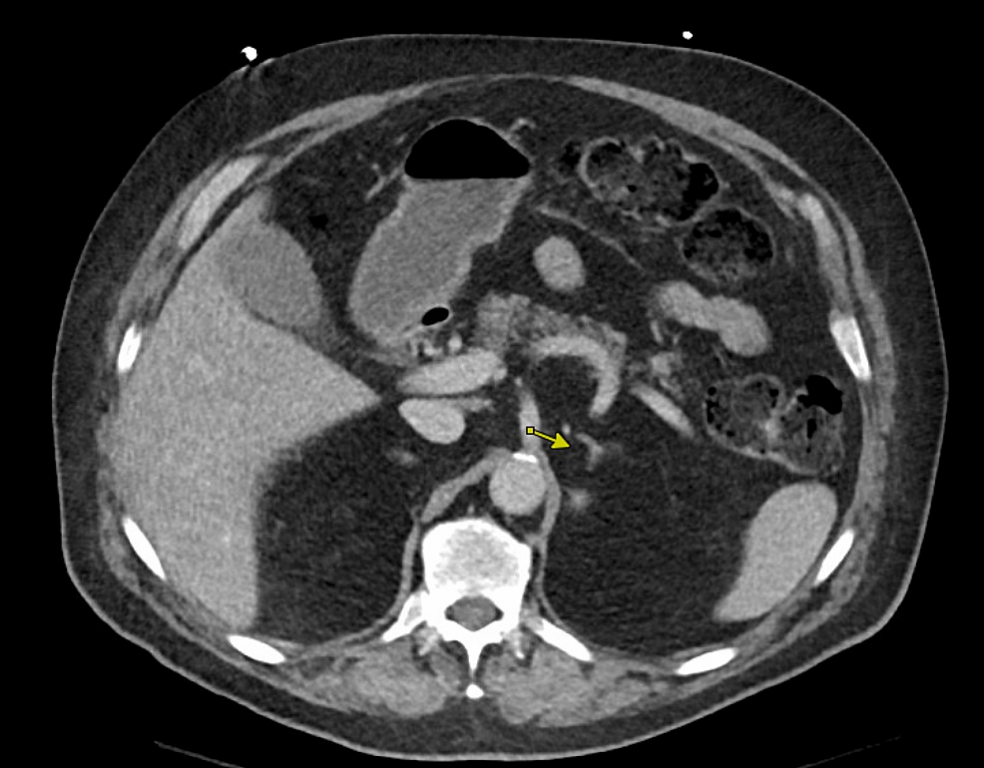
CT imaging of the left adrenal gland (arrow). CT: computed tomography

A 71-year-old man presented to the emergency room with a severe episode of low blood pressure, with systolic blood pressure in the 60s mmHg while undergoing a computed tomography (CT) scan. Prior to admission, he had been experiencing worsening dizziness for a couple of months since his previous hospitalization for chest pain, which was thought to be caused by esophageal spasm as the nuclear stress test did not show any signs of ischemia or prior infarction. The patient experienced dizziness several times a day, with a sensation of near fainting. He also reported fluctuating blood pressure, which ranged from 80 to 180 mmHg. Additionally, he had been sweating more and had lost 15 pounds in two weeks. Upon physical examination, he was found to be extremely pale, and his blood pressure fluctuated between 84/54 mmHg and 210/100 mmHg without having received any medication. The results of his laboratory tests showed an increased WBC count of 14.9 ​​10ˆ3/µL (normal range 4.1-11.1 10ˆ3/µL), high hemoglobin levels of 17.8 g/dL (normal range 13.5-17.5 g/dL), high adjusted calcium levels of 11.6 mg/dL (normal range 8.4-10.2 mg/dL), and high sensitivity troponin levels of 45 pg/mL (normal range 0-20 pg/mL). The EKG revealed sinus rhythm and T wave inversions in the lateral leads. The CT scan showed a 4.6 x 3.9 x 4.3 cm mass in the right upper renal region, indicating primary renal cell neoplasm (Figure 1), and a 3.5 x 3.2 x 3.4 cm enhancing soft tissue lesion adjacent to the superior pole of the left kidney and posterior to the left adrenal gland (Figure 2 and Figure 3). The left-sided mass had an average Hounsfield unit (HU) measurement of 34 without IV contrast, which increased to 91 with IV contrast, and then to 51 on delay images.

While hospitalized, the patient was found to have significant orthostatic hypotension with systolic blood pressure dropping to 77 mmHg and supine hypertension with systolic blood pressure increasing to 210 mmHg. Intravenous hydration was given aggressively. Parathyroid hormone (PTH) and PTH-related protein were checked due to hypercalcemia, which turned out to be normal. In addition to his labile blood pressure, the patient also experienced multiple episodes of chest pain, accompanied by a new-onset left bundle branch block. To assess his cardiac function, an echocardiogram was performed, which revealed normal systolic function without any wall motion or valvular abnormalities. A coronary angiogram was also conducted, which showed that there was no significant obstruction in his coronary arteries. Given the absence of obstructive coronary artery disease, it was thought that his elevated troponin levels were due to demand ischemia. The polycythemia noted on admission was believed to be associated with dehydration, as Erythropoietin (EPO) and JAK2 mutation came back negative.

Based on the patient's labile blood pressure, weight loss, and CT imaging findings, there was a high suspicion of paraganglioma, leading to the discontinuation of beta-blockers and initiation of alpha-adrenergic blockade with terazosin. Later, amlodipine was added for blood pressure control. Several tests were performed, including 24-hour urine metanephrines, urine catecholamines, urine cortisol, plasma metanephrines, renin, and aldosterone, but none were available at the time of discharge. The patient's blood pressure was stabilized with the administration of alpha-blockers and amlodipine, and his condition was deemed stable for him to be discharged from the hospital. 

During the patient's outpatient follow-up, there were remarkable elevations in his urine normetanephrine at 2455 mcg/24 hours (normal range 122-676/24 hours) and plasma normetanephrine at 1325 pg/mL (normal range <= 148 pg/mL). Terazosin was replaced with doxazosin given that doxazosin has a longer half-life. Propranolol was started one week prior to tumor removal for beta-adrenergic blockade after the alpha-adrenergic blockade had been considered sufficient. The patient underwent laparoscopic resection of the left retroperitoneal tumor, and the pathology was consistent with paraganglioma, sympathetic type. Months later, the patient received a CT-guided biopsy of the right renal lesion, and the cytopathology showed oncocytoma.

## Discussion

Paraganglioma diagnosis typically involves measuring urinary metanephrines, urine catecholamines, and/or plasma metanephrines [3]. However, in a community healthcare setting, obtaining the results of these tests can take weeks, which may lead to a delay in the diagnosis and appropriate treatment. This case presented with several symptoms, including intermittent dizziness, chest pain, and fluctuating blood pressure, which can be caused by the periodic surges of catecholamines secreted by paragangliomas. When the CT scan revealed a left-sided retroperitoneal renal lesion, paraganglioma became one of the primary differential diagnoses. On the other hand, the presence of a right renal lesion suggested a primary renal cell neoplasm, leading to the consideration of a metastatic lesion from the renal cell neoplasm instead of paraganglioma, which is relatively rare. To prevent a potential catecholamine surge, the biopsy was avoided as paraganglioma was suspected. In light of the high clinical suspicion of paraganglioma, the alpha-adrenergic blockade was initiated before receiving the results of the biochemical tests. The patient responded well to the treatment and was eventually discharged while prescribed alpha-blockers.

The patient presented with a cluster of symptoms and laboratory abnormalities that can be directly or indirectly associated with paraganglioma. The initial workup for polycythemia using EPO and JAK 2 mutation tests was negative, and aggressive hydration normalized the elevated hemoglobin levels. Therefore, dehydration could explain the elevated hemoglobin level. Hypercalcemia could be attributed to dehydration as well since the PTH and PTH-related protein levels were normal. Due to elevated troponins, chest pain, and new onset left bundle branch block, a coronary angiogram was performed, but it did not indicate obstructive coronary artery disease. The elevated troponin levels on admission were most likely caused by demand ischemia due to fluctuating blood pressures.

The diagnosis of possible paragangliomas in a community healthcare setting can be challenging due to several factors. In addition to the long duration for lab results to return, imaging studies that could be helpful in differentiating paragangliomas, such as metaiodobenzylguanidine (MIBG) scanning and 111-In pentetreotide (OctreoScan) are not available in our facility.

The preferred and only curative treatment option for localized catecholamine-secreting paragangliomas is surgical resection, either through open or laparoscopic surgery, with laparoscopic surgery being favored if feasible [1]. To prevent perioperative cardiovascular complications from catecholamine surges, preoperative pharmacologic preparation is suggested [3]. In our case, the patient denied any family history of neuroendocrine tumors. Genetic mutations are associated with up to 40% of paragangliomas, and this proportion continues to increase as new genes are discovered [2,4]. Thus, further genetic testing should be discussed as is recommended for all patients with paragangliomas [1,3,4]. The most common genes linked to paragangliomas include SDHB, SDHD, VHL, RET, and NF1 [4].

The right renal lesion noted on CT imaging was eventually diagnosed as oncocytoma, which is a rare type of renal cell neoplasm, accounting for 3-7% of renal tumors [5]. Oncocytomas are generally benign and rarely associated with metastases [6]. Coexisting paraganglioma and renal oncocytoma are very uncommon, with limited literature available on the subject [7,8]. A case of renal oncocytoma with SDHB mutation was reported [9], but the association of the genetic predisposition remains unclear. As such, interval follow-up and genetic counseling are warranted in this circumstance.

## Conclusions

This case highlights the challenges of diagnosing and managing paraganglioma in a community healthcare setting. Although this patient's clinical presentations and imaging findings suggested paraganglioma, the delayed return of biochemical studies posed a challenge. In this scenario, it may be reasonable to initiate alpha-adrenergic blockades followed by beta-adrenergic blockades even without a definitive diagnosis to prevent a catecholamine surge when the tumor is manipulated during surgery given that the suspicion for paraganglioma is high. With the coexistence of paraganglioma and renal oncocytoma, interval follow-up and genetic counseling are essential for the patient.

## References

[1] Martucci VL, Pacak K (2014). Pheochromocytoma and paraganglioma: diagnosis, genetics, management, and treatment. Curr Probl Cancer.

[2] Chen H, Sippel RS, O'Dorisio MS, Vinik AI, Lloyd RV, Pacak K (2010). The North American Neuroendocrine Tumor Society consensus guideline for the diagnosis and management of neuroendocrine tumors: pheochromocytoma, paraganglioma, and medullary thyroid cancer. Pancreas.

[3] Lenders JW, Duh QY, Eisenhofer G (2014). Pheochromocytoma and paraganglioma: an endocrine society clinical practice guideline. J Clin Endocrinol Metab.

[4] Lenders JWM, Kerstens MN, Amar L (2020). Genetics, diagnosis, management and future directions of research of phaeochromocytoma and paraganglioma: a position statement and consensus of the Working Group on Endocrine Hypertension of the European Society of Hypertension. J Hypertens.

[5] Kuroda N, Toi M, Hiroi M, Shuin T, Enzan H (2003). Review of renal oncocytoma with focus on clinical and pathobiological aspects. Histol Histopathol.

[6] Dechet CB, Bostwick DG, Blute ML, Bryant SC, Zincke H (1999). Renal oncocytoma: multifocality, bilateralism, metachronous tumor development and coexistent renal cell carcinoma. J Urol.

[7] Bahrami A, Truong LD, Shen SS, Krishnan B (2009). Synchronous renal and adrenal masses: an analysis of 80 cases. Ann Diagn Pathol.

[8] Daskalopoulos G, Delakas D, Charoulakis N, Gourtsoyiannis N, Cranidis A (1996). Coexisting non-functioning pheochromocytoma and renal oncocytoma: a case report and review of the literature. Eur J Radiol.

[9] Henderson A, Douglas F, Perros P, Morgan C, Maher ER (2009). SDHB-associated renal oncocytoma suggests a broadening of the renal phenotype in hereditary paragangliomatosis. Fam Cancer.

